# An association between K65R and HIV-1 subtype C viruses in patients treated with multiple NRTIs

**DOI:** 10.1093/jac/dkx091

**Published:** 2017-04-03

**Authors:** Erasmus Smit, Ellen White, Duncan Clark, Duncan Churchill, Hongyi Zhang, Simon Collins, Deenan Pillay, Caroline Sabin, Mark Nelson, Alan Winston, Sophie Jose, Anna Tostevin, David T. Dunn, on behalf of the UK HIV Drug Resistance Database and the UK Collaborative HIV Cohort

**Affiliations:** 1 Public Health Laboratory Birmingham, Public Health England, Heartlands Hospital, Birmingham, UK; 2 MRC CTU at UCL, University College London, London, UK; 3 Barts and The London NHS Trust, London, UK; 4 Brighton and Sussex University Hospitals NHS Trust, Brighton, UK; 5 Public Health Laboratory Cambridge, Public Health England, Addenbrooke’s Hospital, Cambridge, UK; 6 HIV i-Base, London, UK; 7 Research Department of Infection, Division of Infection and Immunity, University College London, London, UK; 8 Wellcome Trust Africa Centre for Health and Population Sciences, University of KwaZulu Natal, Mtubatuba, South Africa; 9 Research Department of Infection and Population Health London, University College London, London, UK; 10 Chelsea and Westminster Hospital, London, UK; 11 Section of Infectious Diseases, Department of Medicine, Imperial College London, London, UK

## Abstract

**Objectives:** HIV-1 subtype C might have a greater propensity to develop K65R mutations in patients with virological failure compared with other subtypes. However, the strong association between viral subtype and confounding factors such as exposure groups and ethnicity affects the calculation of this propensity. We exploited the diversity of viral subtypes within the UK to undertake a direct comparative analysis.

**Patients and methods:** We analysed only sequences with major IAS-defined mutations from patients with virological failure. Prevalence of K65R was related to subtype and exposure to the NRTIs that primarily select for this mutation (tenofovir, abacavir, didanosine and stavudine). A multivariate logistic regression model quantified the effect of subtype on the prevalence of K65R, adjusting for previous and current exposure to all four specified drugs.

**Results:** Subtype B patients (*n *=* *3410) were mostly MSM (78%) and those with subtype C (*n *=* *810) were mostly heterosexual (82%). K65R was detected in 7.8% of subtype B patients compared with 14.2% of subtype C patients. The subtype difference in K65R prevalence was observed irrespective of NRTI exposure and K65R was frequently selected by abacavir, didanosine and stavudine in patients with no previous exposure to tenofovir. Multivariate logistic regression confirmed that K65R was significantly more common in subtype C viruses (adjusted OR = 2.02, 95% CI = 1.55–2.62, *P *<* *0.001).

**Conclusions:** Patients with subtype C HIV-1 have approximately double the frequency of K65R in our database compared with other subtypes. The exact clinical implications of this finding need to be further elucidated.

## Introduction

K65R is the signature mutation associated with tenofovir resistance^1^ and also confers significant cross-resistance to abacavir, didanosine and stavudine.^2–4^ For reasons that are not entirely clear, it seems that the genetic barrier to K65R development is not as low as with some other NRTIs and NNRTIs. It is well established that the M184V mutation develops quickly in patients failing on emtricitabine/lamivudine, which is not the case for K65R even though it requires only a single nucleotide A–G change at reverse transcriptase (RT) codon 65 to cause the lysine to arginine amino acid change. This A–G point mutation happens regardless of the viral subtype.

The low prevalence of K65R seen in clinical trials and in various resistance databases would support the premise that K65R does not develop that easily in settings where virological failure is well controlled. Worryingly a recent worldwide multicentre retrospective cohort study in patients with treatment failure on tenofovir and NNRTI-based regimens found the K65R prevalence to range from below 20% in Europe and North America to 50% in sub-Saharan Africa.^5^ The wide variation in K65R prevalence is most likely explained by different standards of care, with slower switching after virological failure allowing for greater accumulation of drug resistance to all classes. However, the influence of subtype, which is mostly inextricably linked to demographics, cannot be excluded.

Subtype C viruses are more likely to develop K65R mutations in patients with virological failure than other HIV-1 subtypes. *In vitro* experiments show that the mutation is more easily selected during serial passage than with other subtypes.^6^ There is also strong mechanistic evidence for the facilitated development of the K65R mutation based on the viral template in the codon 64, 65 and 66 RT region found in subtype C.^7^ Some retrospective cohort studies that have looked at factors associated with the emergence of K65R have identified subtype C to be a predictive factor.^8^^,^^9^

However, additional data to support the reported subtype-dependent selection of K65R is needed especially if it can clearly distinguish between the influence of subtype, exposure groups and ethnicity.

The UK national database is well placed to differentiate between these factors as it collects diverse HIV resistance and subtype data typical of the UK HIV epidemic and the aim of this study was to undertake a direct comparative analysis and to determine whether K65R is detected more frequently in subtype C viruses at virological failure.

These questions are important given the global distribution of subtype C and the widespread use of tenofovir in first-line combinations.^10^

## Patients and methods

The UK HIV Drug Resistance Database (UKHDRD) has collated the vast majority of genotypic resistance tests conducted in the UK since the assay was first introduced as part of routine clinical care. Partial *pol* sequences (encoding the protease gene and at least codons 34–234 of RT) generated by Sanger sequencing are transferred electronically from participating laboratories, which use a variety of commercial or in-house assays. A quality assurance programme, in which all laboratories participate, is carried out annually. Tests in the present analysis were conducted between 1996 and 2012. As the absence of any major International AIDS Society (IAS)–USA 2013 list^11^ mutation in a resistance test implies that therapeutic failure was due to lack of drug pressure, such tests were considered uninformative and not included in the analysis.

Patients were considered eligible for the analysis if the following criteria were met: (i) clinical care was received at a centre participating in the UK Collaborative HIV Cohort (UK CHIC) Study (detailed clinical and demographic data, including a complete ART history, are provided by these centres to which resistance test results are regularly linked); (ii) at least one (non-WT) resistance test had been conducted after ART initiation; (iii) K65R had not been detected in any tests conducted prior to ART initiation; and (iv) viral subtype could be assigned based on the nucleotide sequence from a resistance test (see section below).

If the K65R mutation was ever detected (including as a mixture with WT) in an individual patient, the complete ART history until the first sample with this mutation was considered; if K65R was never detected, the patient’s complete ART history until the last sample was considered. ART history was summarized in terms of indicator variables reflecting current and previous (not current) exposure to specific NRTIs, and current and past exposure to the NNRTI and PI classes in general. Exposure was defined as taking a drug for a minimum of 30 days cumulatively, and current exposure was defined as taking the drug at the time of the resistance test or having stopped within the previous 14 days. The specific NRTIs were chosen *a priori* on the basis that they were known to select for K65R (tenofovir, abacavir, didanosine and stavudine)^1–4^^,^^12^^,^^13^ or to protect against the development of K65R (zidovudine) due to antagonistic mutational interactions.^14^^,^^15^

### Viral subtype

Subtypes were defined using the Rega 2 subtyping tool.^16^^,^^17^ Sequences with an unassigned subtype were excluded from the analysis. The propensity for subtype C to develop K65R appears to be due to polymorphisms at positions 64 and 65 rather than any other subtype characteristic.^18^ Subtypes F2, H, CRF07_BC and CRF08_BC^19^ share the same codon usage as subtype C at these positions and have been grouped with subtype C for this analysis. All other subtypes have a B-like codon usage. These were grouped together, but were not combined with subtype B (creating a non-B/C category), as the demographic characteristics of these patients were distinct from those infected with subtype B virus.

### Statistical analysis

Multivariate logistic regression models were fitted to assess the association between viral subtype and the detection of K65R, adjusting for ART history as described above. Interaction terms between subtype and current NRTI exposure were fitted to examine if the effect of subtype on the likelihood of detection of K65R depended on the specific NRTI being prescribed; subtype B and subtypes non-B/C were grouped for this analysis. A sensitivity analysis adjusting for demographic factors (exposure group and ethnicity) in the main effects model was carried out.

All analyses were performed in STATA version 13.1.

## Results

In total, 5100 patients were eligible for analysis, of whom 3410 were infected with a subtype B virus, 810 with a subtype C virus and 880 with a non-B/C subtype virus. In terms of demographic characteristics, the subtype C and non-B/C groups were similar, comprising mainly black heterosexuals, whereas subtype B patients were predominantly white MSM (Table 1). On average, subtype B patients were diagnosed with HIV and started ART several years earlier than patients infected with other subtypes, and had higher viral load and CD4 counts prior to ART initiation, presumably reflecting earlier diagnosis.

**Table 1:** Baseline characteristics by viral subtype

Characteristic	Subtype		
	C^a^ (*n*=810)	B (*n*=3410)	non-B/C (*n*=880)
Year at ART initiation, median (IQR)	2002 (1999–2005)	1997 (1995–2000)	2000 (1997–2004)
Year at HIV diagnosis, median (IQR)	2001 (1997–2004)	1994 (1990–1998)	1999 (1996–2004)
Age at ART initiation (years), median (IQR)	34 (30–40)	34 (29–39)	34 (29–39)
Viral load at ART initiation (copies/mL)^b,^^c^, median (IQR)	38 900 (2100–204 100)	75 700 (11 000–256 200)	51 900 (3200–199 000)
CD4 count at ART initiation (cells/mm^3^)^b^, median (IQR)	134 (46–230)	200 (96–319)	143 (40–232)
Ethnicity, *n* (%)			
white	71 (8.8)	2688 (78.8)	85 (9.7)
black	605 (74.7)	183 (5.4)	647 (73.5)
Asian	20 (2.5)	53 (1.6)	18 (2.0)
other	35 (4.3)	209 (6.1)	24 (2.7)
unknown	79 (9.8)	277 (8.1)	106 (12.0)
Exposure group, *n* (%)			
MSM	39 (4.8)	2650 (77.7)	35 (4.0)
heterosexual-males	241 (29.8)	174 (5.1)	282 (32.0)
heterosexual-females	423 (52.2)	145 (4.3)	444 (50.5)
IVDU	3 (0.4)	170 (5.0)	14 (1.6)
other	30 (3.7)	76 (2.2)	28 (3.2)
unknown	74 (9.1)	195 (5.7)	77 (8.8)

a Subtype C category includes subtypes F2, H, CRF07_BC and CRF08_BC  (see the Patients and methods section).

b Parameters measured less than 6 months before ART initiation.

c Rounded to nearest 100.

Table 2 shows exposure to specific antiretroviral drugs by viral subtype. Reflecting the earlier date of ART initiation, patients infected with subtype B virus were more likely to have received first-generation NRTIs (zidovudine, stavudine, didanosine) and less likely to have received tenofovir. This group also had slightly greater exposure to PIs and lesser exposure to NNRTIs. Patterns for the subtype C and subtype non-B/C groups were broadly similar, except that the latter group had a higher frequency of exposure to stavudine and to PIs.

**Table 2:** ART exposure by viral subtype

ART exposure	Subtype		
	C (*n*=810), *n* (%)	B (*n*=3410), *n* (%)	non-B/C (*n*=880), *n* (%)
TDF			
previous	75 (9.3)	271 (7.9)	97 (11.0)
current	309 (38.1)	1032 (30.3)	339 (38.5)
ever	384 (47.4)	1303 (38.2)	436 (49.5)
ABC			
previous	118 (14.6)	481 (14.1)	145 (16.5)
current	210 (25.9)	797 (23.4)	220 (25.0)
ever	328 (40.5)	1278 (37.5)	365 (41.5)
ddI			
previous	155 (19.1)	1015 (29.8)	216 (24.5)
current	113 (14.0)	886 (26.0)	161 (18.3)
ever	268 (33.1)	1901 (55.7)	377 (42.8)
d4T			
previous	180 (22.2)	1224 (35.9)	240 (27.3)
current	61 (7.5)	854 (25.0)	106 (12.0)
ever	241 (29.8)	2078 (60.9)	346 (39.3)
ZDV			
previous	302 (37.3)	1687 (49.5)	393 (44.7)
current	233 (28.8)	830 (24.3)	213 (24.2)
ever	535 (66.0)	2517 (73.8)	606 (68.9)
PI			
previous	126 (15.6)	837 (24.5)	163 (18.5)
current	298 (36.8)	1421 (41.7)	393 (44.7)
ever	424 (52.3)	2258 (66.2)	556 (63.2)
NNRTI			
previous	247 (30.5)	1019 (29.9)	304 (34.5)
current	352 (43.5)	1247 (36.6)	339 (38.5)
ever	599 (74.0)	2266 (66.5)	643 (73.1)

TDF, tenofovir disoproxil fumarate; ABC, abacavir; ddI, didanosine; d4T, stavudine; ZDV, zidovudine.

Overall, K65R was detected in 446 (8.7%) patients. Note that this value reflects cumulative incidence (mutation ever detected) rather than cross-sectional prevalence, and is related to the duration of drug selection pressure and the number of resistance tests performed. The frequency of K65R was highest among patients infected with a subtype C virus (14.2%; 115/810), approximately double that observed for subtype B (7.8%; 267/3410) and subtype non-B/C (7.3%; 64/880).

**Figure 1:**
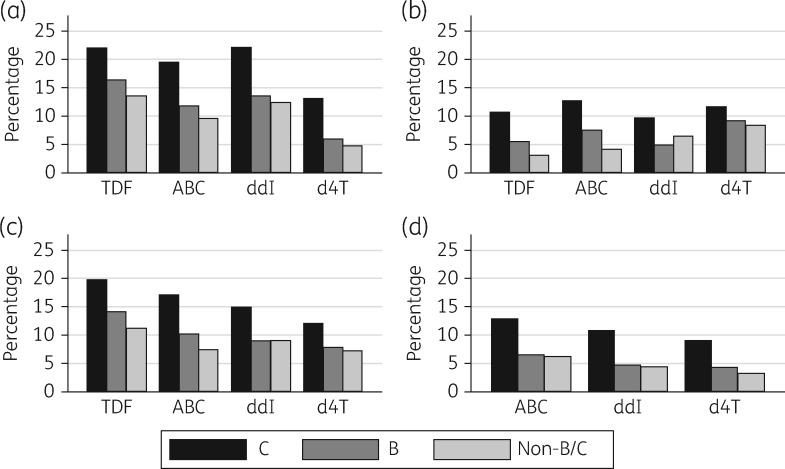
Proportion of patients ever detected with K65R by exposure to selected NRTIs. (a) Current exposure. (b) Previous exposure. (c) Any exposure. (d) Current exposure among patients who never received tenofovir. TDF, tenofovir disoproxil fumarate; ABC, abacavir; ddI, didanosine; d4T, stavudine.

The association between K65R detection and viral subtype was consistently observed regardless of current or previous exposure to the NRTIs that select for K65R (Figure 1). However, it is not possible to draw conclusions about the relative selective pressure exerted by different NRTIs from this univariate analysis since patients received many different permutations of the drugs and as K65R may have been selected on a single occasion or multiple occasions. Among patients who had never taken tenofovir, the drug that selects most strongly for K65R, the mutation remained commonly observed and the subtype difference persisted (Figure 1d).

Table 3 shows the results of the multivariate logistic regression analysis, which adjusts for individual patient drug exposure. This analysis confirmed the highly significant difference in the frequency of K65R among subtype C patients compared with subtype B patients (adjusted OR = 2.02, 95% CI = 1.55–2.62, *P *<* *0.001). There was no evidence of a difference between subtype non-B/C and subtype B viruses (adjusted OR = 0.89, 95% CI = 0.66–1.21, *P *=* *0.47). The strongest drug selection pressure was observed for tenofovir, with patients who were taking this drug at the time of the resistance test being 5.03-fold (95% CI = 3.90–6.50) more likely to have developed K65R compared with patients who had never taken tenofovir. Current exposure to abacavir and didanosine were also significant predictors of K65R, but no effect of exposure to stavudine (either previous or current) was observed. As expected, patients taking zidovudine were less likely (adjusted OR = 0.41, 95% CI = 0.29–0.59) to develop K65R. Those currently on an NNRTI were at an increased risk of developing the mutation (adjusted OR = 1.78, 95% CI = 1.30–2.44) whereas those currently on a boosted PI were protected (adjusted OR = 0.29, 95% CI = 0.21–0.40). With the exception of tenofovir, previous drug exposure had no effect on the development of K65R, which reflects the low replicative capacity of this mutation. The sensitivity analysis, additionally controlling for ethnicity and exposure group, gave very similar results, but with larger standard errors around parameter estimates: the adjusted ORs for subtype C and subtype non-B/C (relative to subtype B) were 2.04 (95% CI = 1.35–3.08) and 0.89 (95% CI = 0.57–1.38), respectively (Table 4).

**Table 3:** Logistic regression analysis of detection of K65R by viral subtype and ART exposure

	Total	K65R, *n* (%)	OR	aOR^a^	95% CI	*P*
Subtype						<0.001
B	3410	267 (7.8)	1.00	1.00	—	
C	810	115 (14.2)	1.95	2.02	1.55–2.62	<0.001
non-B/C	880	64 (7.3)	0.92	0.89	0.66–1.21	0.47
TDF exposure						<0.001
never	2977	137 (4.6)	1.00	1.00	—	
past	443	26 (5.9)	1.29	2.09	1.29–3.40	
current	1680	283 (16.8)	4.20	5.03	3.90–6.50	
ABC exposure						<0.001
never	3129	233 (7.4)	1.00	1.00	—	
past	744	57 (7.7)	1.03	0.97	0.69–1.37	
current	1227	156 (12.7)	1.81	2.06	1.62–2.63	
ddI exposure						<0.001
never	2554	202 (7.9)	1.00	1.00	—	
past	1386	79 (5.7)	0.70	0.97	0.69–1.37	
current	1160	165 (14.2)	1.93	2.66	2.00–3.53	
d4T exposure						0.81
never	2435	229 (9.4)	1.00	1.00	—	
past	1644	153 (9.3)	0.99	0.93	0.69–1.25	
current	1021	64 (6.3)	0.64	0.89	0.61–1.29	
ZDV exposure						<0.001
never	1442	187 (13.0)	1.00	1.00	—	
past	2382	208 (8.7)	0.64	0.80	0.62–1.04	
current	1276	51 (4.0)	0.28	0.41	0.29–0.59	
PI exposure						<0.001
never	1862	224 (12.0)	1.00	1.00	—	
past	1126	128 (11.4)	0.94	0.84	0.63–1.11	
current	2112	94 (4.5)	0.34	0.29	0.21–0.40	
NNRTI exposure						<0.001
never	1592	68 (4.3)	1.00	1.00	—	
past	1570	109 (6.9)	1.67	1.12	0.79–1.59	
current	1938	269 (13.9)	3.61	1.78	1.30–2.44	

TDF, tenofovir disoproxil fumarate; ABC, abacavir; ddI, didanosine; d4T, stavudine; ZDV, zidovudine.

a Adjusted OR from main effects model.

**Table 4:** Logistic regression analysis of detection of K65R by viral subtype and demographic factors

	Total	K65R, *n* (%)	OR	aOR^a^	95% CI	*P*
Subtype						<0.001
B	3410	267 (7.8)	1.00	1.00	—	
C	810	115 (14.2)	1.95	2.04	1.35–3.08	0.001
non-B/C	880	64 (7.3)	0.92	0.89	0.57–1.38	0.60
Ethnicity^b^						0.35
white	2844	217 (7.6)	1.00	1.00	—	
black	1435	153 (10.7)	1.44	1.28	0.88–1.85	
Asian	91	5 (5.5)	0.70	0.69	0.27–1.80	
other	268	18 (6.7)	0.87	0.85	0.50–1.45	
Exposure^b^						0.12
MSM	2724	222 (8.1)	1.00	1.00	—	
heterosexual-males	697	75 (10.8)	1.36	1.00	0.66–1.52	
heterosexual-females	1012	92 (9.1)	1.13	0.68	0.45–1.04	
IVDU	187	15 (8.0)	0.98	1.05	0.58–1.90	
other	134	8 (6.0)	0.72	0.53	0.24–1.17	

a Adjusted OR from main effects model, adjusting for drug exposure and other factors in table.

b Unknown categories included in model, but excluded from table and calculation of *P*.

**Figure 2:**
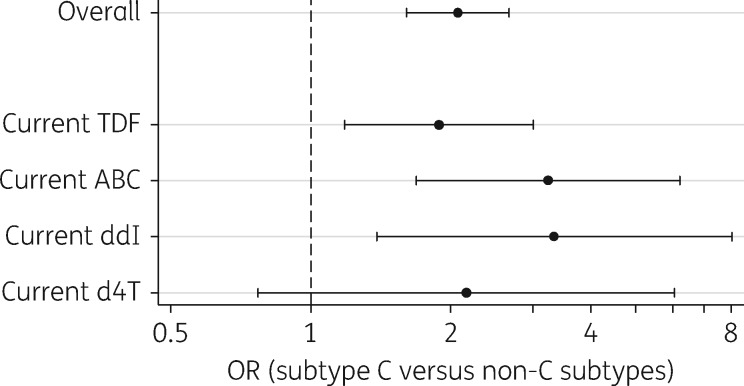
Likelihood of K65R mutation in subtype C viruses versus all other subtypes by current NRTI exposure. Estimates derived from extended logistic regression model including interaction terms. TDF, tenofovir disoproxil fumarate; ABC, abacavir; ddI, didanosine; d4T, stavudine.

We added interaction effects between subtype and current NRTI exposure to examine if the subtype C effect was drug dependent. In this analysis subtype B and subtypes non-B/C were combined to constrain the number of extra parameters. A significant association between subtype C and an increased risk of K65R was observed with tenofovir, abacavir and didanosine. This effect was not found to be significant amongst those currently on stavudine due to a smaller number of K65R mutations occurring in this group. The OR was lower for tenofovir (OR = 1.89, 95% CI = 1.18–3.01) than the other NRTIs, although a test for statistical heterogeneity was not significant (*P *=* *0.59) (Figure 2).

## Discussion

Our analysis provides strong clinical evidence that there is an increased risk of finding the K65R mutation among subtype C viruses following virological failure. Our results are in keeping with previous reports and confirm that patients with subtype C are twice as likely as those with other subtypes to develop a K65R mutation.^8^ It is difficult to determine the exact influence subtype has on the propensity of K65R development in patients with virological failure due to the fact that subtype C is linked to certain demographic factors such as ethnicity, immigration, culture, lifestyle and socioeconomic status. Our study is large enough to control for this bias because we have a large group of patients with non-B/C subtypes with similar demographics and baseline characteristics to the patients infected with subtype C viruses in the sense that this group also consisted largely of black heterosexual patients (Table 1). It is therefore reasonable to assume that the same factors that could influence adherence, such as socioeconomic factors, were broadly the same for the non-B/C and subtype C groups of patients even though they may have come from different countries before residing in the UK. Furthermore, a separate sensitivity analysis (Table 4) that additionally controlled for ethnicity and exposure group confirmed the association between subtype C and K65R.

Various other factors have also been shown to increase the risk of K65R development such as current tenofovir and/or NNRTIs,^9^ dual didanosine + tenofovir therapy,^8^^,^^20^ low starting CD4 count and length of virological failure.^5^^,^^21^ Zidovudine and/or current boosted PI-containing therapy is known to reduce the risk.^9^ As with other studies, data from this study show that patients infected with subtype B viruses were more likely to have been exposed to zidovudine, have an earlier calendar year of starting ART (less exposure to tenofovir and more exposure to zidovudine), have higher CD4 counts and are more likely to be currently on PIs. Looking at other factors associated with the emergence of K65R, as reported by von Wyl *et al.*,^9^ we also found that current zidovudine or boosted PI-containing therapy conferred protection (adjusted OR = 0.41 and 0.29, respectively). The risk of K65R developing was increased on NNRTI-based therapy (adjusted OR = 1.78) and the highest risk was observed with current treatment with tenofovir (adjusted OR = 5.03). Of interest, we also observed that K65R was common amongst patients who had never been prescribed tenofovir (Figure 2).

There is generally a low overall prevalence of K65R reported in various resistance databases.^20^^,^^22^^,^^23^ The Swiss HIV Cohort Study (SHCS) found a cumulative prevalence of 2.2% amongst patients on a tenofovir-containing regimen with at least one genotypic resistance test.^9^ The prevalence is higher when data from patients who have recently failed ART is analysed and in the aforementioned study it goes up to 10.1% in patients on tenofovir. Data analysis from the UK CHIC Study, which is a representative cohort of the UK HIV population on ART, shows the prevalence of K65R to be 13.2% in those failing a tenofovir- and efavirenz-based regimen.^21^ There is a further increase in K65R prevalence in developing countries in patients who fail a non-zidovudine-containing NRTI regimen. This is especially the case in sub-Saharan Africa where subtype C viruses predominate.^24–27^ A recent multicentre retrospective cohort study (TenoRes) that combined data from cohorts and clinical trials across 36 countries found K65R prevalence rates of more than 50% in sub-Saharan African patients with treatment failure on tenofovir- and NNRTI-based regimens.^5^ The wide variation in the worldwide prevalence of K65R is most likely explained by different standards of care, most notably the length of time spent on failing ART, but our data indicate that subtype C *per se* contributes to the high prevalence seen in countries where subtype C is prevalent. The OR of 1.95 we observed is roughly in keeping with the ORs observed in the TenoRes cohort study^5^ (OR = 2.44) and the EuResist consortium study (OR = 2.22),^8^ which confirms that subtype C is significantly associated with a higher probability of K65R emergence.

Our data are consistent with previous evidence that subtype C is a strong risk factor for the development of K65R in patients with virological failure. The clinical implication may therefore be that patients who are infected with subtype C viruses are at an increased risk of virological failure because of the propensity of the virus to develop a K65R mutation when on a tenofovir-based regimen. This seems to be supported by two recent studies.^28^^,^^29^ However, when we studied this hypothesis using UK CHIC data, we found that although subtype C and subtype non-B/C viruses have virological failure rates twice as high as patients with subtype B viruses, the difference disappeared when adjusted for demographic and clinical characterises.^30^ An explanation for the differences in treatment response and risk of K65R developing between subtypes could be that a single point mutation under an optimal treatment setting does not affect outcome. However, when other factors that lead to differential non-adherence, such as demographics and clinical characteristics, are present then subtype C viruses have a greater propensity to develop a K65R mutation.^31^

Small retrospective retreatment cohort studies suggested that second-line therapy is as successful with K65R as when the mutation is not present, usually with zidovudine in the regimen. The strongest predictor of virological response was the addition of zidovudine to the retreatment regimen.^32^ However, the high prevalence of K65R in low-income countries where K65R presence can be as high as 50% has led to increasing use of zidovudine in second-line therapy. Further second-line studies are needed in order to establish if and when it is safe to remove zidovudine and possibly re-use tenofovir + lamivudine/emtricitabine when the viral load has become fully suppressed and the individual is on a boosted PI.

A further implication of this study is that pre-exposure prophylaxis (PrEP) could potentially be compromised in countries where subtype C viruses are common. So far, PrEP studies using tenofovir ± emtricitabine have reported a low incidence of K65R acquisition when failing tenofovir-based PrEP, even when more sensitive sequencing is employed.^33^ The influence of subtype C on the likelihood of K65R development has not yet been studied in patients with PrEP failure due to the low incidence of breakthrough infections and resistance in clinical trial settings. Our data would suggest a possible increased risk of acquiring K65R in patients with subtype C virus who fail PrEP, especially in settings where PrEP is not stopped soon after seroconversion. K65R resistance also tends to disappear within a month of stopping ART^34^ and therefore more sensitive baseline resistance testing may need to be performed in patients with a history of PrEP usage without adequate monitoring. The role of emtricitabine/lamivudine in dual-therapy PrEP might also mitigate against the risk of early resistance to tenofovir. It still has to be established if minority variant K65R substantially increases a patient’s risk of virological failure in a resource-limited setting.^35^

A strength of this study is the study size, which makes it the largest comparative study to date, with more patients than the EuResist consortium study.^8^ A shortcoming of many studies is the strong association between viral subtype, exposure groups and ethnicity, which are difficult to differentiate. The subtype diversity of the epidemic in the UK made it possible to compare subtype C viruses with non-B/C subtype viruses that share similar demographics and treatment history, but which have different viral templates.

This study included patients with a number of different NRTI exposure histories, which included some of the older NRTIs, which could be considered a weakness of the study. However, this would not have unduly influenced the results as this study mainly explored the hypothesis that the subtype C template has a predilection for developing a K65R mutation. A further consideration is the fact that the K65R mutation could have disappeared due to the adverse effect it has on replication fitness of the virus upon stopping combination ART and could therefore be underrepresented in our database. It is also possible that K65R existed as a minority variant prior to therapy as a result of undetected transmitted drug resistance, but the possibility of this is small and should not have affected our results.

In conclusion, our analysis shows that patients with subtype C HIV-1 have approximately double the frequency of K65R in our database compared with other subtypes. The exact clinical implications of this finding need to be further elucidated.
